# Delphinidin, One of the Major Anthocyanidins, Prevents Bone Loss through the Inhibition of Excessive Osteoclastogenesis in Osteoporosis Model Mice

**DOI:** 10.1371/journal.pone.0097177

**Published:** 2014-05-13

**Authors:** Sawako Moriwaki, Keiko Suzuki, Masashi Muramatsu, Atsushi Nomura, Fumihide Inoue, Takeshi Into, Yuji Yoshiko, Shumpei Niida

**Affiliations:** 1 Laboratory of Genomics and Proteomics, National Center for Geriatrics and Gerontology (NCGG), Aichi, Japan; 2 Biobank Omics Unit, National Center for Geriatrics and Gerontology (NCGG), Aichi, Japan; 3 Department of Pharmacology, School of Dentistry, Showa University, Tokyo, Japan; 4 Nihon Seiyaku Kogyo, Co., Ltd., Aichi, Japan; 5 Department of Oral Bacteriology, Division of Oral Infections and Health Sciences, Asahi University School of Dentistry, Gifu, Japan; 6 Department of Oral Growth and Developmental Biology, Hiroshima University Graduate School of Biomedical Sciences, Hiroshima, Japan; National Center for Cell Science, India

## Abstract

Anthocyanins, one of the flavonoid subtypes, are a large family of water-soluble phytopigments and have a wide range of health-promoting benefits. Recently, an anthocyanin-rich compound from blueberries was reported to possess protective property against bone loss in ovariectomized (OVX) animal models. However, the active ingredients in the anthocyanin compound have not been identified. Here we show that delphinidin, one of the major anthocyanidins in berries, is a potent active ingredient in anti-osteoporotic bone resorption through the suppression of osteoclast formation. *In vitro* examinations revealed that delphinidin treatment markedly inhibited the differentiation of RAW264.7 cells into osteoclasts compared with other anthocyanidins, cyanidin and peonidin. Oral administration of delphinidin significantly prevented bone loss in both RANKL-induced osteoporosis model mice and OVX model mice. We further provide evidence that delphinidin suppressed the activity of *NF-κB*, *c-fos*, and *Nfatc1*, master transcriptional factors for osteoclastogenesis. These results strongly suggest that delphinidin is the most potent inhibitor of osteoclast differentiation and will be an effective agent for preventing bone loss in postmenopausal osteoporosis.

## Introduction

Osteoporosis, characterized by low bone mineral density (BMD) and fragility of the bone matrix, is a complex bone disease with various causes, including aging, estrogen deficiency and genetics, and leads to increased fracture risk, especially in the elderly population. Current anti-osteoporosis drugs such as bisphosphonates have been widely used and their bone protective effects are well established. However, many individuals with osteoporosis remain undiagnosed, untreated and at risk of fracture because of the lack of symptoms and public awareness [1]. Hence, a dietary nutritional approach is important for primary prevention of bone loss.

Natural compounds in fruits and vegetables such as polyphenols have a wide range of biological and pharmacological effects [2]–[5]. Several flavonoids, a subgroup of polyphenols including hesperidin, quercetin and luteolin, have been shown to possess preventive efficacy for bone loss in ovariectomized (OVX) animal models [6]–[8]. Most showed potent suppressive effects on the differentiation and/or function of osteoclasts, bone resorbing multinucleated giant cells [7]–[10]. Osteoclasts are formed by the fusion of mononuclear preosteoclasts derived from monocyte-macrophage linage cells. Osteoclast differentiation is tightly regulated by two key molecules, receptor activator of NF-κB ligand (RANKL), a TNF super-family cytokine, produced by osteoblasts, and its receptor RANK, which are expressed on osteoclast precursors [11]. Several flavonoids seem to be inhibitors of RANKL-RANK signaling-related molecules, including NF-κB and nuclear factor for activated T cells (NFATc1), master osteoclastogenic molecules located downstream of NF-κB [7]–[10].

In this study, we focused on delphinidin, one of the aglycone nuclei of anthocyanins, as an active agent for anti-bone loss *in vivo*. Anthocyanins are a large family of water-soluble red/blue/purple flavonoid pigments in fruits and vegetables, and have a wide range of health-promoting benefits, for example, as anti-oxidants. Many studies have demonstrated the pharmacological effects of anthocyanidins on human diseases, including night blindness, cancer, obesity and heart diseases [12]–[16]. Recently, a blueberry extract, an anthocyanin-rich compound, was reported to possess protective properties against bone loss in OVX rats [17]. Moreover, it has been reported that anthocyanins extracted from bilberry and blackcurrant exerted potent inhibitory effects on NF-κB activation in activated monocytes, and consequently led to the downregulation of pro-inflammatory mediators [18]. The NF-κB signaling pathway is well established to be critical for osteoclastogenesis. Commonly, extracted anthocyanin compounds consist of several kinds of anthocyanins derived from plural aglycone bases such as cyanidin, delphinidin and peonidin, major anthocyanidins in extracts of berries, including blueberry. However, to our knowledge, the active ingredients for bone metabolism have not been identified. To address this question is useful for not only developing the high efficient dietary supplements but also finding novel drug seeds. Here we show that delphinidin is the most potent inhibitor of osteoclastogenesis and will be an effective agent for preventing *in vivo* bone degradation.

## Materials and Methods

### Anthocyanins and Anthocyanidins

Bilberon-25, a concentrated extract of bilberry, was purchased from Tokiwa Phytochemical Co., Ltd. (Chiba, Japan). Cassis extract-35, a concentrated extract of blackcurrant, was generously donated by Tama Biochemical Co., Ltd. (Tokyo, Japan). Cyanidin chloride (C_15_H_11_ClO_6_), delphinidin chloride (C_15_H_11_ClO_7_) and peonidin chloride (C_16_H_13_ClO_6_) were from Extrasynthése (Lyon, France) and Sigma-Aldrich (St Louis, MO, USA), respectively. Epicatechin (C_15_H_14_O_6_) was purchased from Kurita Analysis Service Co., Ltd. (Ibaraki, Japan).

### Cell Culture

RAW264.7 cells, a mouse macrophage cell line, were used as osteoclast precursor cells and maintained in α modified essential medium (α-MEM) supplemented with 10% fetal bovine serum (FBS) at 37°C and 5% CO_2_. For osteoclast induction, cells were plated in a 96-well plate at a density of 4×10^3^ cells/well and stimulated with 100 ng/ml RANKL for 4 days. For the inhibition study, cells were pre-incubated in α-MEM supplemented with vehicle or with various concentrations of anthocyanin-rich extracts and anthocyanidins, 1 h before the addition of RANKL. To confirm multinucleated osteoclast formation, the cultured cells were fixed in 10% formalin for 3 minutes, and then stained with an osteoclast marker enzyme, tartrate-resistant acid phosphatase (TRAP). Effects of anthocyanins and anthocyanidins on osteoclast formation were evaluated by morphological observations and the intensity of TRAP staining was measured at 520 nm using a spectrophotometer (SpectraMax M5; Molecular Devices, Sunnyvale, CA, USA).

Osteoblasts were isolated from newborn calvariae of C57BL/6J mice, as described previously with slight modifications [19]. Briefly, calvariae were minced and sequentially digested with collagenase solution at 37°C. Cells retrieved from the osteogenic cell fractions were separately cultured in α-MEM supplemented with 10% FBS and antibiotics. After 24 h, cells were pooled and grown in multi-well plates in the same medium containing 50 µg/ml of ascorbic acid (AA), 10 µM dexamethasone (Dex) and 10 mM β-glycerophosphate (β-GP) with or without anthocyanin-rich extracts. After two weeks culture, cells were stained with *von* Kossa’ s staining to determine the matrix mineralization, as described previously [19].

### Animals and Treatments

To assess the protective effect of delphinidin on *in vivo* bone loss, we created soluble RANKL (sRANKL)-induced osteoporosis model mice, which were established by Yasuda and his colleagues [20]. Seven-week-old female C57BL/6 mice (n = 17) were purchased from CLEA Japan (Tokyo, Japan). Mice were divided into three groups: control mice (cont, n = 5), osteoporosis model mice (vehicle, n = 6) and delphinidin-treated osteoporosis mice (Del, n = 6). Twelve mice were intraperitoneally injected with GST-RANKL (1 mg/kg; Oriental Yeast Co., Ltd., Kyoto, Japan) twice at interval of 3 days. First injection was performed at 3 days after starting of delphinidin-treatment. For six mice, delphinidin treatment (10 mg/kg/day) via gavage started 3 days before the first injection of GST-RANKL, and continued for 14 days. Another six mice received the same volume of vehicle, a mixture of dimethyl sulfoxide (DMSO) and water.

We further assessed the effect of delphinidin using OVX mice. Eight-week-old female C57BL/6 mice were purchased from Charles River Laboratory Japan (Kanagawa, Japan). Thirty mice were either sham-operated (n = 6) or OVX (n = 24). OVX mice were divided into four groups (n = 6×4): OVX control, low-dose delphinidin (1 mg/kg), intermediate-dose delphinidin (3 mg/kg), and high-dose delphinidin (10 mg/kg) groups. After OVX, delphinidin was administrated orally, as mentioned above, for 28 days. OVX-control mice received the same volume of vehicle, a mixture of DMSO and water.

All mice were housed in an animal room (temp, 22±2°C; humidity, 50%; light/dark cycle, 12 h) with free access to food and water. At the end of treatment, the mice were sacrificed. Femora were removed and fixed with 3.7% formaldehyde in phosphate-buffered saline solution (pH7.4) for 16 h. After rinsing, bones were immersed in 80% ethanol and stored at 4°C in a refrigerator. These experiments were approved by the Animal Experimental Committees of Showa University and NCGG, respectively.

### Microcomputed Tomography

Bone morphometric parameters and microarchitectual properties of the femur were determined using a microcomputed tomography (µCT) system (inspeXio SMX-90CT; Shimadzu Co., Kyoto, Japan) with X-ray tube settings of 90 kV and 108 µA. The femur was placed in a microcentrifuge tube filled with PBS and scanned at a voxel size of 15.0 µm. TRI/3D-BON software (RATOC System Engineering Co., Tokyo, Japan) was used to generate 3D models from 272 2D-transverse slices, and bone morphometry was performed in the distal metaphyseal region (500 µm in length) of femora. For quantitative analysis, BV/TV, trabecular thickness, trabecular number, trabecular separation and trabecular spacing were determined using TRI/3D-BON software.

### Histomorphometric Analysis of Bone

Femora were fixed in 70% ethanol and embedded in glycolmethacrylate (GMA) without decalcification. Serial sections were cut and stained with Villanueva bone stain for bone histomorphometry, as previously reported [21]. The histomorphometorical analysis was performed at the Ito Bone Science Institute (Niigata, Japan).

### Extraction of Nuclear Protein and Assessment of NF-κB Activation

Nuclear cell lysates were prepared using a Nuclear Extract Kit (Active Motif, Tokyo, Japan) following the manufacturer’s instructions. We examined the inhibitory effect of delphinidin on NF-κB activation in RAW264.7 cells. Briefly, cells were pretreated with delphinidin or vehicle for 1 h, and subsequently stimulated with sRANKL. After 3 h of stimulation, cells were washed with ice-cold PBS(−) and hypotonic buffer added from the kit to explore the nucleus. To dissolve the nuclear protein, complete lysis buffer containing 1 mM DTT and protease inhibitor cocktail were added to the cell lysate precipitation and the concentration of protein was measured using Prostain (Active Motif). Cell lysates equal to the concentration were measured by TransAM NF-κB p65 Chemiluminescence (Active Motif), which is a high-throughput assay to quantify NF-κB activation, that is, binding activity to DNA-binding fragments fixed to the plate bottom.

### Reverse Transcriptase-polymerase Chain Reaction Analysis

For reverse transcriptase-polymerase chain reaction (RT-PCR) assays, total RNA was prepared using an AurumTotal RNA Mini Kit (Bio-Rad, Richmond, CA, USA) according to the manufacturer’s instructions. One microgram of total RNA from each sample was reverse-transcribed with Ready-To-Go You-Prime First-Strand Beads (GE Healthcare, Piscataway, NJ, USA). cDNA was amplified by PCR. The PCR products were electrophoresed on a 1.2% agarose gel.

### Quantitative Real-time PCR Analysis

Quantitative real-time PCR (qPCR) assays were performed with the CFX96 Real-Time system (Bio-Rad) using the Thunderbird SYBR qPCR Mix (Toyobo, Osaka, Japan). Expression levels were normalized to glyceraldehyde-3-phosphate dehydrogenase (GAPDH).

### Statistical Analysis

All data are the mean ± standard deviation (SD). Statistical analyses were performed using unpaired Student t tests. *P*<0.05 was considered significant.

## Results

### Effects of Anthocyanin on Osteoclastogenesis and Osteogenesis

**Figure 1:**
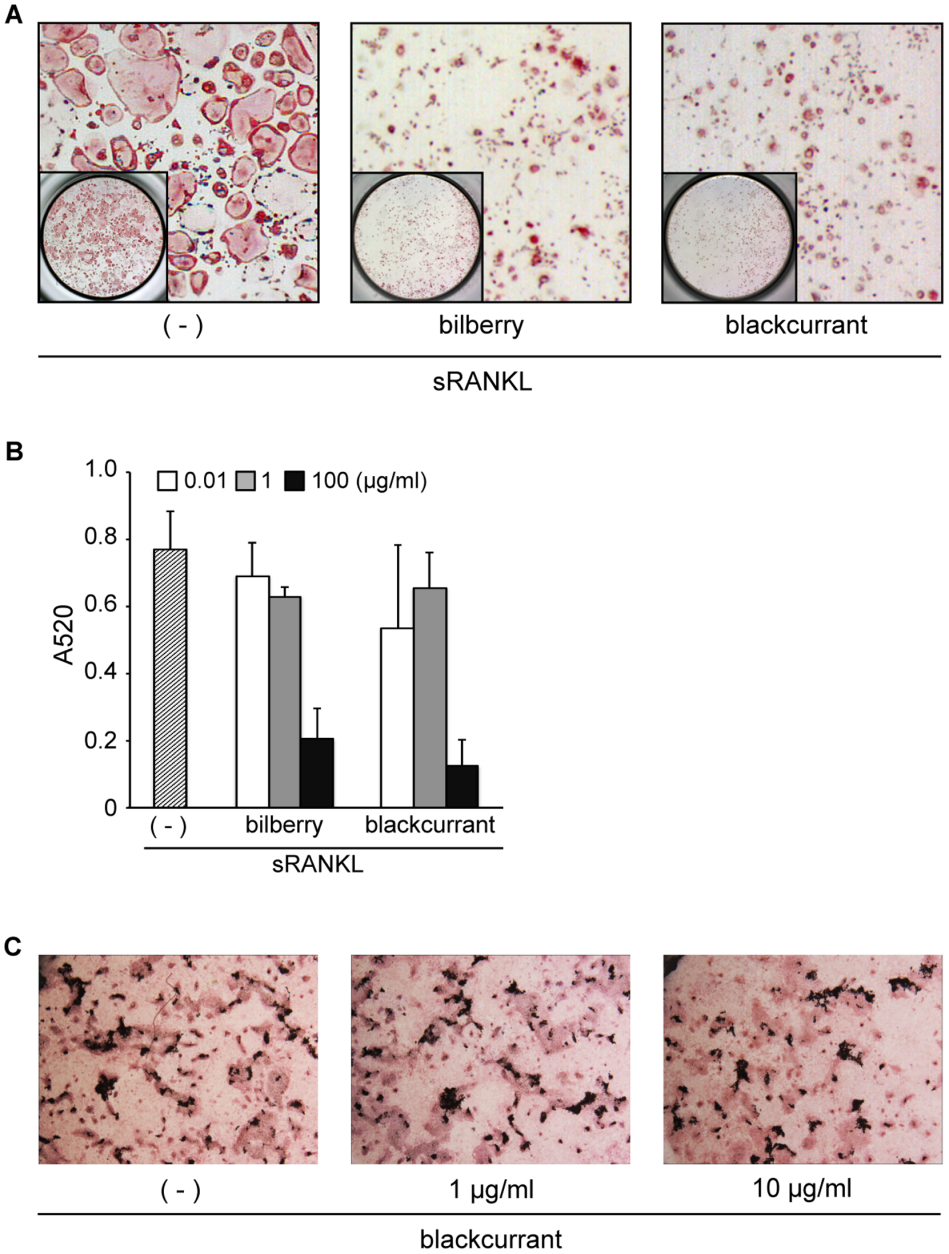
Anthocyanin-rich compounds extracted from bilberry and blackcurrants inhibit *in vitro* osteoclastogenesis, but not osteogenesis. RAW264.7 cells were pre-incubated for 1 h in the presence of various concentrations of anthocyanin compounds, and subsequently cultured for 4 days in the presence of 100 ng/ml RANKL. Cells were fixed and stained with TRAP. A: Numerous TRAP-positive multinucleated giant cells, osteoclasts, were induced by RANKL treatment (left panel). In contrast, large osteoclasts were significantly decreased by addition of anthocyanin compounds (middle and right panels). B: Anti-osteoclastogenic activity of anthocyanin compound was evaluated by the absorption ratio of the red-stained area per well at 520 nm on a spectrometer. C: Primary osteoblastic cells were cultured in the medium containing 50 µg AA, 10 µM Dex and 10 mM β-GP with or without anthocyanin compound for 2 weeks. No significant effect was observed on the osteoblast differentiation and mineral apposition.

Initially, we tested the effects of anthocyanin compound extracted from bilberry (*Vaccinium myrtillus* L.) and blackcurrant (*Ribes nigrum*) on osteoclast formation using RAW264.7 cells as osteoclast precursors. The number of TRAP-positive multinucleated giant cells, osteoclasts, differentiated from sRANKL-stimulated RAW246.7 cells, was markedly decreased by pretreatment with both extracts, suggesting that both anthocyanin compounds contain active ingredients to suppress osteoclast formation (Figure 1A and 1B). In contrast, no significant effect of anthocyanin compound (blackcurrant) was found on mineral apposition (Figure 1C). These results indicated that target cell of anthocyanin may be osteoclasts in bone metabolism.

### Effects of Anthocyanidins on Osteoclastogenesis

**Figure 2:**
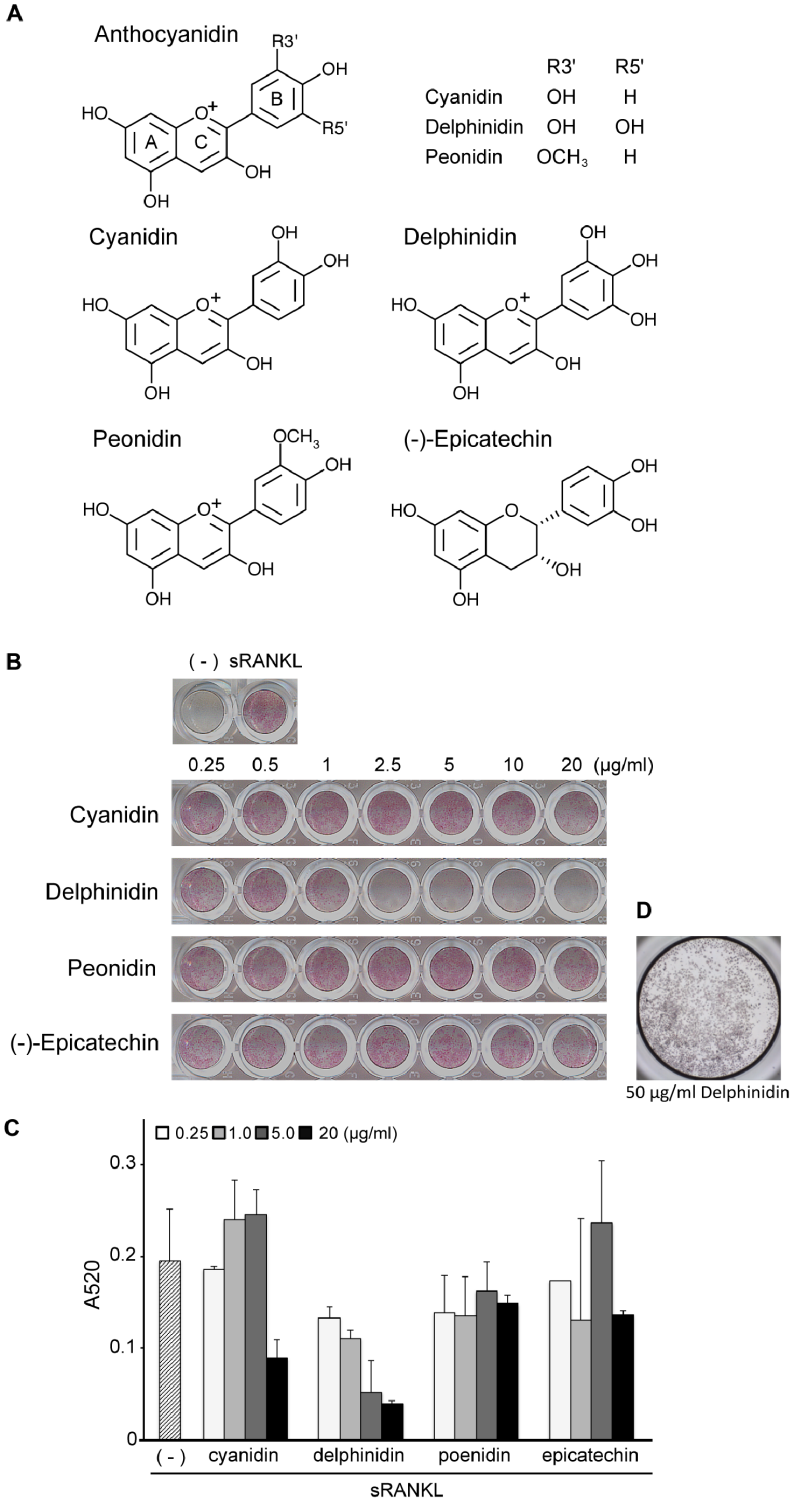
Effects of various anthocyanidins on *in vitro* osteoclastogenesis. A: The structure of tested three anthocyanidins and (−)-epicatechin as a negative control. B: RAW264.7 cells were pretreated for 1 h with increasing concentrations (0.25–20 µg/ml) of three anthocyanidins and epicatechin, and cultured for 4 days in the presence of RANKL (100 ng/ml). Red-stained area in each well indicates osteoclast population. Delphinidin strongly inhibited RANKL-induced osteoclastogenesis at >1 µg/ml. Cyanidin showed moderate inhibition at 20 µg/ml. C: Absorption ratio also supported the results of TRAP-staining observation. D: RAW264.7 cells were cultured with delphinidin alone for 4 days. No cytotoxic effect of delphinidin was found. Cells were stained by crystal violet.

Next, to identify the active ingredients (aglycone bases) as anti- osteoclastogenic factors, we investigated three major anthocyanidins, cyanidin, delphinidin and peonidin, which are commonly contained in many purple berries, including bilberry and blackcurrant (Figure 2A). RAW264.7 cell cultures containing various concentrations of these anthocyanidins were incubated with sRANKL (100 ng/ml) for 4 days. As shown in Figure 2, delphinidin treatment caused a dose-dependent inhibition of osteoclastogenesis. Although cyanidin treatment showed a mild suppressive effect on osteoclastogenesis at a concentration of 20 µg/ml, doses ≤10 µg/ml did not affect osteoclastogenesis. In contrast, peonidin treatment showed no significant effect on osteoclastogenesis, similar to the control compound, (−)-epicatechin. Similar results were also obtained at a higher concentration (50 µg/ml) of anthocyanidins (data not shown). Additionally, we confirmed that delphinidin did not induce cell death in RAW264.7 cell cultures without RANKL (Figure 2D).

### Inhibitory Effects of Dietary Delphinidin on Bone Loss *in vivo*

**Figure 3:**
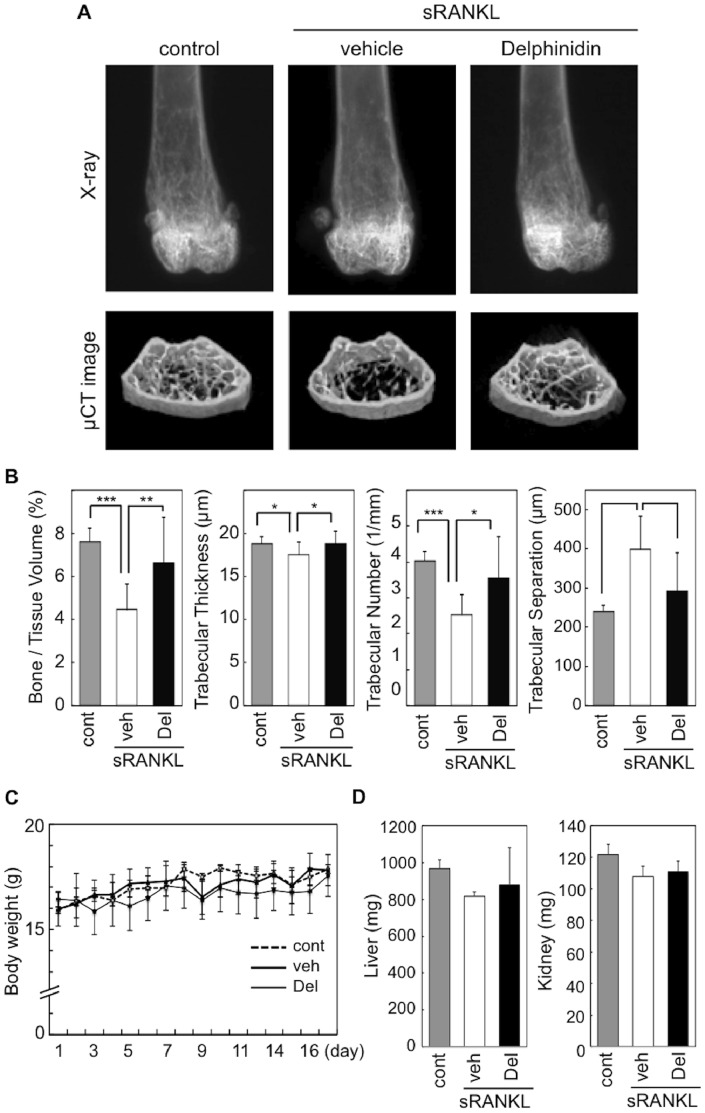
Effect of delphinidin on bone loss in RANKL-induced osteoporosis mice. A: Representative X-ray and microcomputed tomography images of the distal femurs of intact mice (control), RANKL-induced osteoporosis mice (vehicle), and delphinidin-treated RANKL-induced osteoporosis mice (Delphinidin). B: Effect of RANKL injection and delphinidin treatment on trabecular microarchitectural parameters. Values are expressed as the mean ± SD. *p<0.05, **p<0.01, ***p<0.001. C: Comparison of body weight between each experimental group. Values are expressed as the mean ± SD. D: Comparison of weights of liver and kidney in each experimental group. Values are expressed as the mean ± SD.

To determine the relevance of our *in vitro* findings of delphinidin bioactivity to the *in vivo* situation, we investigated the anti-osteoporotic effect of delphinidin on sRANKL-induced osteoporosis model mice. 3D-images and morphometric parameters clearly demonstrated that the cancellous bone volume of femoral distal metaphysis was markedly decreased by sRANKL treatment, compared with intact control mice. In contrast, delphinidin treatment significantly inhibited cancellous bone degradation in sRANKL-treated mice (Figure 3A, B).

All animals were weighed daily for 17 days, and there was no significant change among the experimental groups (Figure 3C). Although liver and kidney weights were slightly decreased in sRANKL-treated mice compared with control mice, these differences were not significant (Figure 3D). Also, there was no significance between sRANKL-treated mice and sRANKL/delphinidin-treated mice (Figure 3C).

**Figure 4:**
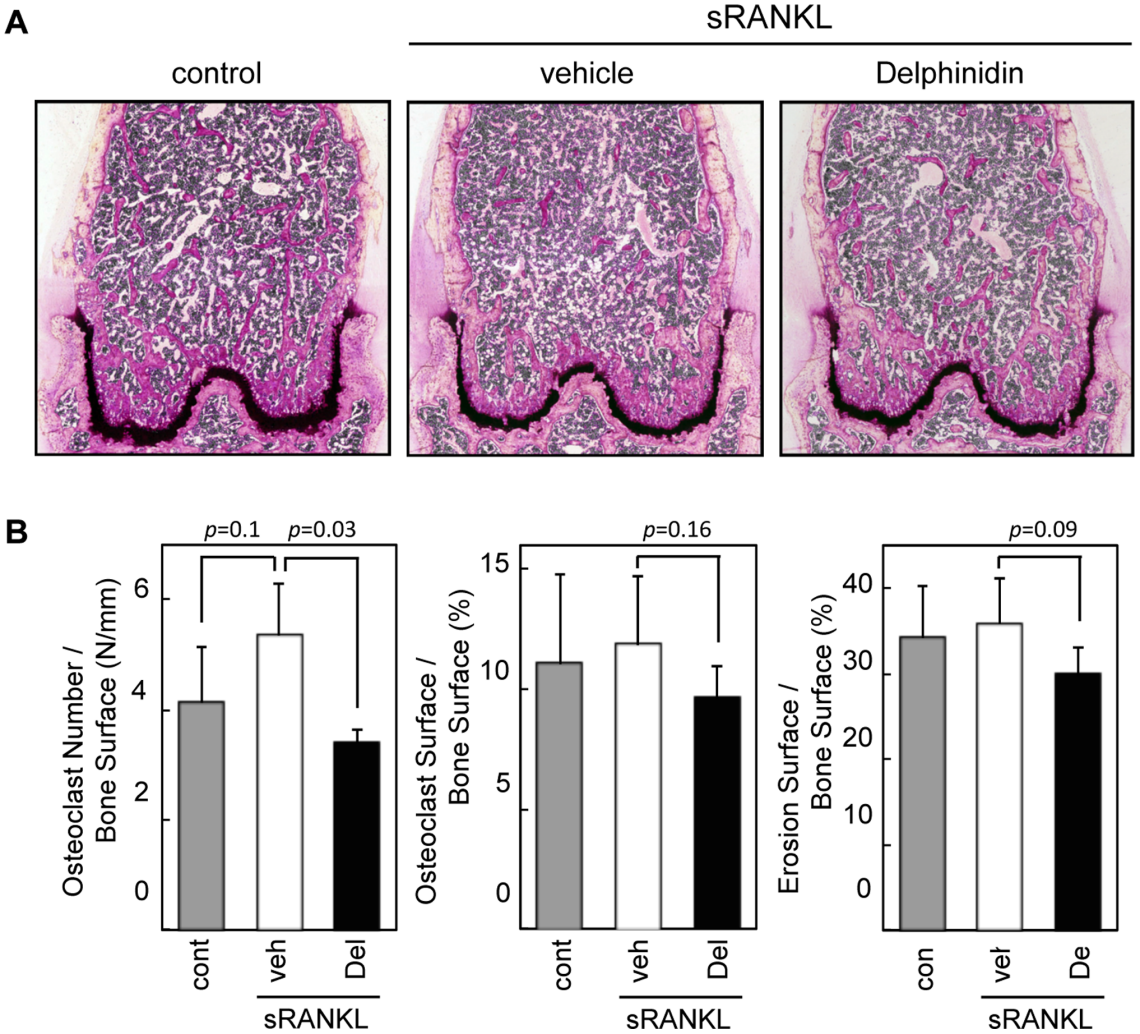
Histomorphometric analysis of anti-bone resorption effect of delphinidin. A: Histological images of distal femurs of intact mice (control), RANKL-induced osteoporosis mice (vehicle), and delphinidin (10 mg/Kg)-treated RANKL-induced osteoporosis mice (Delphinidin). Sections were stained by Villanueva staining. B: Bone-resorption parameters were determined by histomorphometric analysis. Values are expressed as the mean ± SD.

We further performed histomorphometrical observation of bone tissues. Microscopic observation demonstrated that the number of bone trabeculare obviously decreased in sRANKL-treated osteoporotic mice. In contrast, many bone trabeculae remained in the bones of delphinidin-treated mice (Figure 4A). Despite the limited number of bone trabeulare, osteoclast number was significantly decreased (*p* = 0.03) in delphinidin-treated mice than non-treated osteoporotic mice (Figure 4B). Other major two resorption-parameters were also downregulated in the mice administrated delphinidin, although there were no statistically significant differences between these two groups (Figure 4B). Consistent with the *in vitro* examinations, these results suggested that delphinidin acts, at least, on the osteoclast differentiation directly.

**Figure 5:**
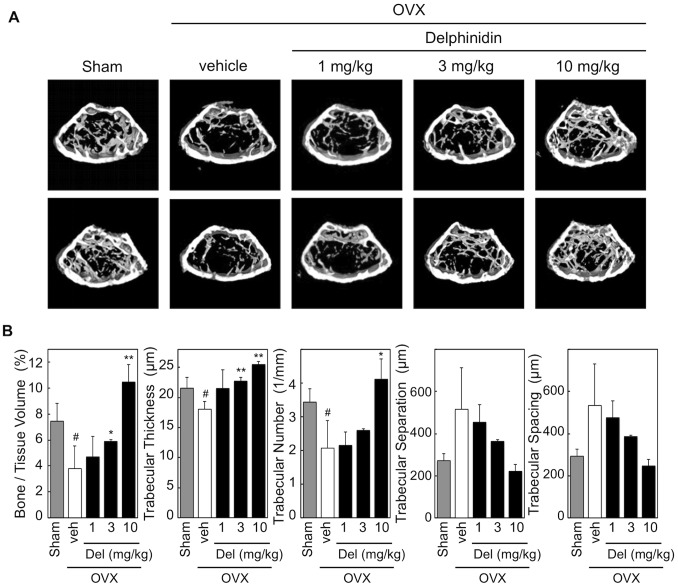
Effect of delphinidin on bone loss in OVX mice. A: Microcomputed tomography images of the distal femurs (axial view of the metaphyseal region) of sham-operated mice, OVX mice (vehicle), and delphinidin-treated OVX mice (Delphinidin). B: Effect of delphinidin on trabecular microarchitectural parameters. Values are expressed as the mean ± SD. *p<0.05, **p<0.01 (vs vehicle), ^#^ *p*<0.05 (vs sham).

We further assessed the anti-bone resorption effect of delphinidin using OVX mice, a standard model of osteoporosis. OVX mice were orally administered delphinidin at three different doses. A significant anti-osteoporotic effect was confirmed in over half the mice treated with not only high-dose (10 mg/kg) but also intermediate-dose (3 mg/kg) delphinidin (Figure 5). In this examination, uterus weight did not change in delphinidin-treated OVX mice (data not shown), suggesting that delphinidin did not act on the uterus like SERMs (selective estrogen receptor modulators) such as isoflavons [22]. Together, our results demonstrated that ingestion of delphinidin acts as a potent anti-osteoporotic agent for preventing osteoporosis.

### Mechanism of Delphinidin-mediated Inhibition of Osteoclastogenesis

**Figure 6:**
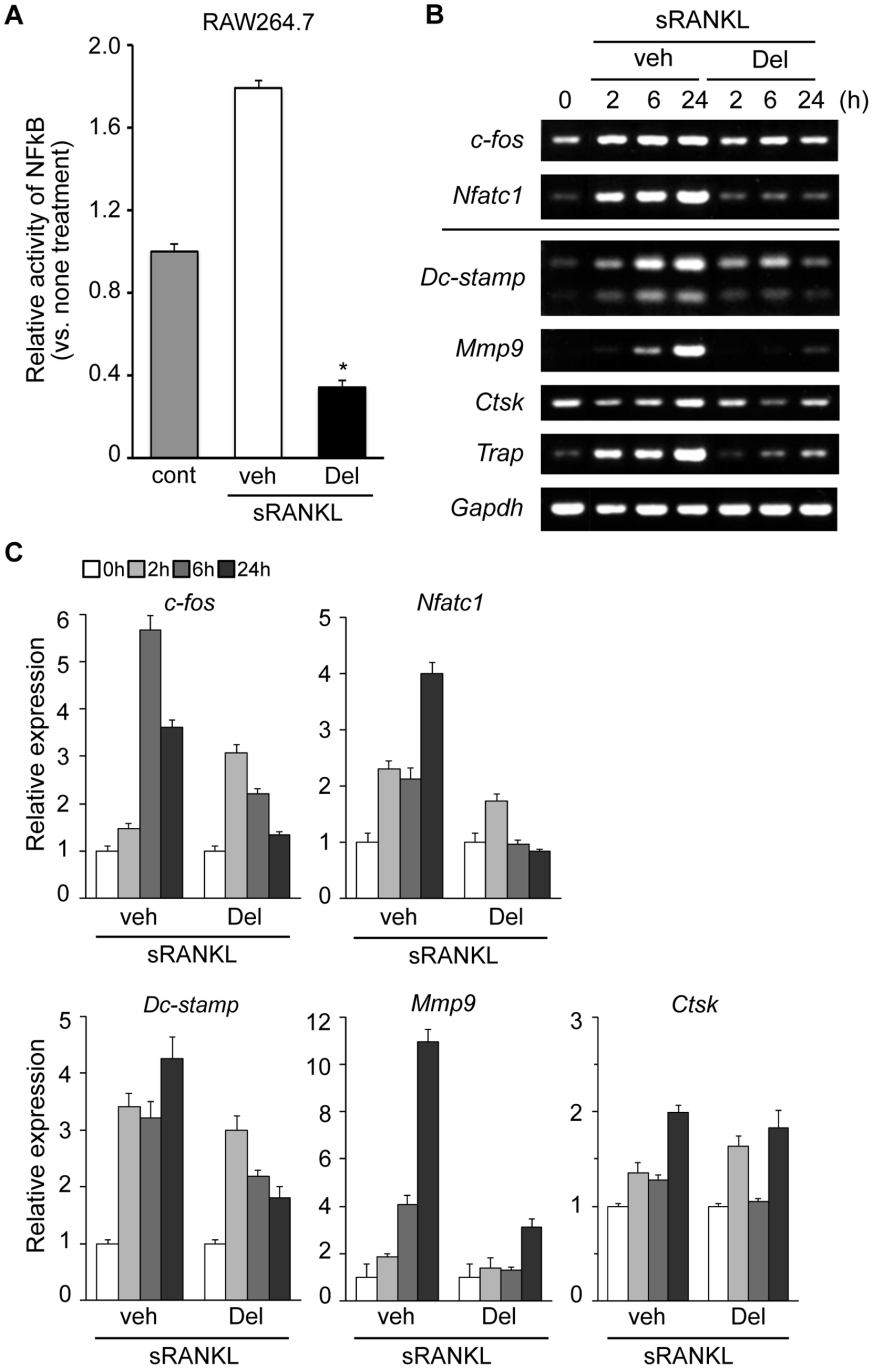
Delphinidin suppresses the expression of major osteoclastogenic molecules in osteoclast precursor cells. (A) Quantification of NF-κB DNA-binding activity in control cells, agent stimulated cells and delphinidin-treated cells. RANKL-stimulated NF-κB DNA-binding activity was significantly inhibited by delphinidin treatment (*p<0.001). (B) RT-PCR and (C) qPCR analysis of the expression of osteoclastogenic transcriptional genes, *c-fos* and *Nfatc1*, and osteoclast marker genes, *Dc-stamp, Mmp9, Ctsk* and *Trap,* in RAW264.7 cells.

Since previous work indicated that anthocyanin extract inhibited NF-κB activation in activated monocytes, we first examined the activation of NF-κB in RAW264.7 cells using the TransAM assay. When the cells were stimulated by sRANKL, the activation of NF-κB p65 subunit was upregulated promptly. In contrast, RANKL-mediated NF-κB activation was markedly downregulated in cells treated by delphinidin, suggesting its effect at a transcriptional level (Figure 6A).

RANKL-induced *c-fos* activation is another pivotal event in the early phase of osteoclastogenesis [11], [23]. NFATc1 is a master transcriptional factor located at the downstream cross-point of both NF-κB and c-Fos pathways in osteoclastogenesis [11], [23]. We therefore investigated whether delphinidin affects the activation of *c-fos* and *Nfatc1* genes. Additionally, we examined the alteration of the expression levels of osteoclast marker genes in delphinidin-treated RAW264.7 cells. Our results demonstrate that RANKL-induced *c-fos* activation was attenuated by delphinidin treatment. Furthermore, expression levels of the *Nfatc1* gene were markedly decreased in delphinidin-treated cells (Figure 6B, C). Consequently, expression levels of osteoclast marker genes, especially *Mmp9* and *Trap*, were reduced by delphinidin treatment (Figure 6B, C). In addition, expression levels of *Dc-stamp*, a fusion protein gene for multinucleation, were significantly decreased in delphinidin-treated cells (Figure 6B, C). Together, these results suggest that the anti-osteoclastogenic effect of delphinidin is clearly associated with alteration of the signaling cascade triggered by RANKL.

## Discussion

Last year, an interesting epidemiological report was published in the Journal of Bone Mineral Research. Welch and his colleagues investigated the association between habitual flavonoid intake with bone mineral density in 3,160 women, and concluded that flavonoid intake, especially anthocyanin (median intake: 13.7 mg/day), was beneficial for bone-protective effects in women [24]. Devareddy *et al*. previously reported that the extracted anthocyanin compound showed a protective property against bone loss in OVX rats [17]. However, the active ingredients in the anthocyanin compound still have not been identified. In the present study, we provide evidence that delphinidin, one of the major anthocyanidins, is a potent preventive natural agent for osteoporosis and might support the conclusion of the epidemiological investigation experimentally.

We evaluated the inhibitory effects of three major anthocyanidins contained in berries on *in vitro* osteoclast formation, and consequently identified that delphinidin was the most potent active ingredient. In our preliminary tests using their glycosides, similar results in terms of potency were obtained (data not shown), suggesting that anti-osteoclastogenic activity depends on the structure of aglycone form. Since anthocyanins are much more stable than other hydrophilic flavonoid glycosides, they are rapidly absorbed into blood as glycosides without changing to aglycones after consumption [25], [26]. Then, we directly administrated delphinidin into the osteoporosis mice, and consequently obtained significant results that delphinidin acts as an inhibitor of bone loss. Generally, aglycones of flavonoids are absorbed more rapidly and in greater amounts than their glycoside forms in human and animals [27], [28]. It is supposed that delphinidin administrated orally was quickly absorbed and delivered to bone tissue. However, *in vivo* kinetics of anthocyanidins (aglycones) have not been much investigated as compared with those of glycosides. To elucidate the mechanisms of absorption and metabolism of the aglycones are important for understanding of bioavailability of anthocyanidins. The results obtained from this study may be useful to investigate the *in vivo* circulation of anthocyanidins in the future.

Consistent with *in vitro* examinations, the increased number of osteoclasts under osteoporotic conditions was significantly decreased by delphinidin treatment, resulting in prevention of bone loss. Delphinidin-mediated suppression of osteoclast formation is likely to be a key event associated with the inhibition of bone resorption in osteoporosis mice, as well as anti-osteoporosis drugs, bisphosphonates. RANK signaling triggered by RANKL modulates the activation of various signal pathways including NF-κB and mitogen-activated protein kinases (MAPKs) via TRAF6, an essential mediator of RANK signaling. Previous studies revealed that anthocyanin compounds or anthocyanidins were involved in several signal pathways in various cell types. Karlsen and his colleagues revealed that anthocyanin inhibited LPS-induced NF-κB activation in monocytes [18]. Hafeez *et al.* indicated that delphinidin inhibited NF-κB signaling at multiple levels in prostate cancer cells, and induced apoptosis [29]. These reports have important implications for our understanding of the mechanism of delphinidin in osteoclastogenesis. Consistent with previous work from other groups, our results demonstrate that delphinidin strongly suppressed the activation of NF-κB in RANKL-stimulated osteoclast precursors, RAW264.7 cells. We also found the downregulation of *c-fos* and *Nfatc1*, pivotal regulators of osteoclastogenesis, in delphinidin-treated cells (Figure 6). It is recognized that the induction of NFATc1 depends on NF-κB and AP-1 containing c-Fos. Karlsen *et al*. further demonstrated that plasma levels of NF-κB-related pro-inflammatory mediators were downregulated in participants receiving anthocyanin supplementation compared with the placebo group [18]. In this context, it is plausible that delphinidin administration limits the number of osteoclasts through the downregulation of osteoclastogenic genes in osteoporotic mice. However, details of the molecular mechanism whereby delphinidin attenuates the activation of these osteoclastogenic regulators are still unclear. For example, the anti-oxidant property of anthocyanidins including delphinidin is one of the possible mechanisms underlying the limited gene expression, as in other cell types [14]–[16]. Reactive oxygen species (ROS) act as second messengers in signal transduction and gene expression in various cell types. In osteoclast precursors, intracellular levels of ROS are transiently increased in response to RANKL, and modulate the activity of the RANKL signaling pathway [30], [31]. Since NF-κB is an oxidative stress-sensitive molecule [32], downregulation of NF-κB might depend on the anti-oxidant activity of delphinidin.

The finding that peonidin failed to suppress *in vitro* osteoclastogenesis is important to clarify the mechanism. This result suggests that anthocyanidins cannot always inhibit osteoclastogenesis. Peonidin is another representative anthocyanidin and is the principal ingredient in, for example, cranberry anthocyanin extracts. Previous work using mouse epidermal cells (JB6 cells) suggested that peonidin exhibited chemopreventive activity through inhibition of the phosphorylation of extracellular signal-regulated kinases (ERKs) in the cells [33]. If so, peonidin possibly alters the phenotype of osteoclast formation because RANKL-mediated activation of MAPKs, including ERKs, would be implicated in osteoclastogenesis [11], [30], [34]. However, there was no influence of peonidin on osteoclast differentiation in our experiments. Another investigation using JB6 cells also mentioned that peonidin treatment could not attenuate MAPK activity, including ERKs, in activated cells [35]. By contrast, cyanidin exhibited a mild suppressive effect on osteoclastogenesis at high concentration (≥20 µg/ml). Bioactivity of anthocyanidins may be characterized according to different substituent groups on the B-ring. As shown in Figure 2A, the C3′ positions (R3′) of the B-ring of cyanidin and delphinidin are hydroxyl (–OH) groups, while that of peonidin is a methoxy (–OCH_3_) group. The ortho-dihydroxyphenyl structure seems to be critical for triggering anti-osteoclastogenic activity among the anthocyanidins. In addition, to clarify the difference whereby delphinidin induces more potent anti-osteoclastogenic activity compared with cyanidin, further studies will be required.

Meanwhile, the bone histomorphometry revealed that delphinidin treatment caused not only decrease of bone resorption but also a slight increase in number of osteoblasts and osteoid volume (no statistical significance). However, in our preliminary tests using the mouse primary osteoblasts, anthocyanin treatment did not stimulate calcification nodule formation (data not shown). Delphinidin consumption may have stimulated concomitantly upregulation of osteogenic potential through the in vivo network. It is desirable that delphinidin induces bone formation simultaneously in osteoporosis. In OVX rats, blueberry diet altered bone formation markers [17]. To investigate regarding the effect of delphinidin on the bone formation may be needed in future.

In summary, this study demonstrated for the first time that delphinidin possesses a potent inhibitory effect against RANKL-mediated osteoclastogenesis via downregulation of osteoclastogenic factors such as *Nfκb*, *c-fos* and *Nfatc1*. Oral administration of delphinidin showed a preventive effect on bone loss in osteoporosis model mice. Our results suggest that dietary delphinidin or anthocyanin supplements containing delphinidin may be useful for the prevention of RANKL-mediated bone loss such as postmenopausal osteoporosis.
